# (1*R*,2*R*,3*S*,6a*S*,7*R*,8*R*,9*S*,12a*S*)-1,2,3,7,8,9-Hexahydroxy­perhydro­dipyrido[1,2-*a*:1′,2′-*d*]pyrazine-6,12-dione

**DOI:** 10.1107/S1600536810009165

**Published:** 2010-03-17

**Authors:** S. F. Jenkinson, D. Best, F. X. Wilson, G. W. J. Fleet, D. J. Watkin

**Affiliations:** a Department of Organic Chemistry, Chemistry Research Laboratory, University of Oxford, Oxford OX1 3TA, England; b Summit PLC, 91 Milton Park, Abingdon, Oxon OX14 4RY, England; c Department of Chemical Crystallography, Chemistry Research Laboratory, University of Oxford, Oxford OX1 3TA, England

## Abstract

The crystal structure of the title compound, C_12_H_18_N_2_O_8_, exists as O—H⋯O hydrogen-bonded layers of mol­ecules running parallel to the *ab* plane. Each mol­ecule is a donor and acceptor for six hydrogen bonds. The absolute stereochemistry was determined by the use of d-glucuronolactone as the starting material.

## Related literature

For the isolation and biological activity of pipecolic acids, see: Manning *et al.* (1985 ▶); di Bello *et al.* (1984 ▶). For the synthesis of pipecolic acids, see: Bashyal *et al.* (1986 ▶); Bashyal, Chow & Fleet (1987 ▶); Bashyal, Chow, Fellows & Fleet (1987 ▶).
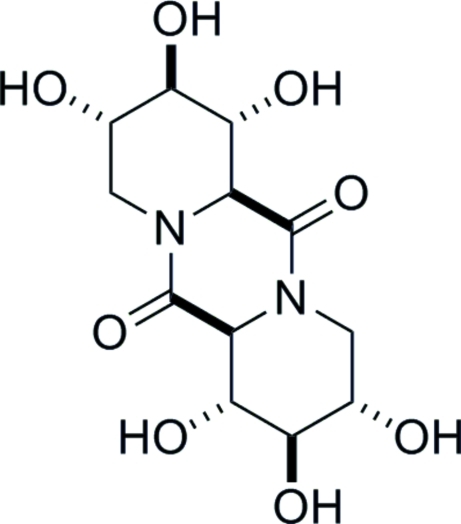


## Experimental

### 

#### Crystal data

C_12_H_18_N_2_O_8_
*M* *_r_* = 318.28
Orthorhombic, 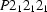

*a* = 7.8711 (2) Å
*b* = 8.1526 (2) Å
*c* = 19.5783 (5) Å
*V* = 1256.34 (5) Å^3^
*Z* = 4
Mo *K*α radiation
μ = 0.14 mm^−1^
*T* = 150 K
0.40 × 0.10 × 0.10 mm

#### Data collection

Nonius KappaCCD area-detector diffractometer
Absorption correction: multi-scan (*DENZO*/*SCALEPACK*; Otwinowski & Minor, 1997 ▶) *T*_min_ = 0.85, *T*_max_ = 0.99
12748 measured reflections
1663 independent reflections
1348 reflections with *I* > 2σ(*I*)
*R*_int_ = 0.061

#### Refinement

*R*[*F* ^2^ > 2σ(*F* ^2^)] = 0.042
*wR*(*F* ^2^) = 0.101
*S* = 0.93
1662 reflections
199 parameters
H-atom parameters constrained
Δρ_max_ = 0.35 e Å^−3^
Δρ_min_ = −0.43 e Å^−3^

### 

Data collection: *COLLECT* (Nonius, 2001 ▶); cell refinement: *DENZO*/*SCALEPACK* (Otwinowski & Minor, 1997 ▶); data reduction: *DENZO*/*SCALEPACK*; program(s) used to solve structure: *SIR92* (Altomare *et al.*, 1994 ▶); program(s) used to refine structure: *CRYSTALS* (Betteridge *et al.*, 2003 ▶); molecular graphics: *CAMERON* (Watkin *et al.*, 1996 ▶); software used to prepare material for publication: *CRYSTALS*.

## Supplementary Material

Crystal structure: contains datablocks global, I. DOI: 10.1107/S1600536810009165/lh5005sup1.cif

Structure factors: contains datablocks I. DOI: 10.1107/S1600536810009165/lh5005Isup2.hkl

Additional supplementary materials:  crystallographic information; 3D view; checkCIF report

## Figures and Tables

**Table 1:** Hydrogen-bond geometry (Å, °)

*D*—H⋯*A*	*D*—H	H⋯*A*	*D*⋯*A*	*D*—H⋯*A*
O8—H81⋯O17^i^	0.83	1.95	2.756 (4)	162
O22—H221⋯O1^ii^	0.83	2.22	2.917 (4)	141
O19—H191⋯O11^iii^	0.82	2.12	2.793 (4)	139
O11—H111⋯O13^iv^	0.83	1.86	2.685 (4)	173
O17—H171⋯O8^iii^	0.80	1.87	2.633 (4)	157
O6—H61⋯O19^v^	0.83	1.97	2.680 (4)	143

Symmetry codes: (i) 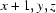; (ii) 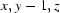; (iii) 
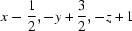
; (iv) 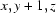; (v) 
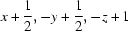
.

**Fig. 1.**
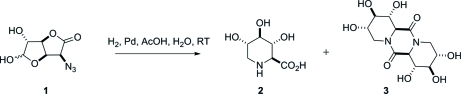
Synthetic Scheme.

**Fig. 2.**
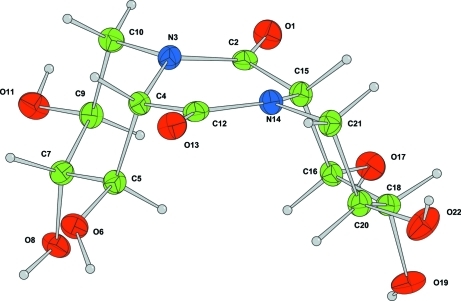
The title compound with displacement ellipsoids drawn at the 50% probability level. H atoms are shown as spheres of arbitary radius.

**Fig. 3.**
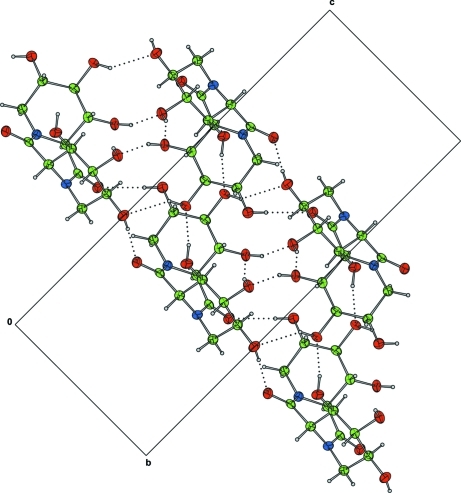
Packing diagram for the title compound projected along the a-axis. Hydrogen bonds are shown by dotted lines.

**Fig. 4.**
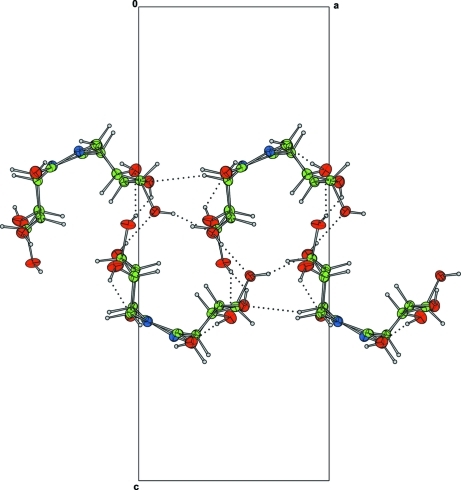
Packing diagram for the title compound projected along the b axis. Hydrogen bonds are shown by dotted lines.

## supplementary crystallographic information

### Comment

2*S*,3*R*,4*R*,5*S*-Trihydroxypipecolicacid (BR1) 2 (Fig.1), a sugar mimic of glucuronic acid, has been isolated from the seeds of Baphia racemosa (Manning *et al.*, 1985) and shown to inhibit both glucuronidase and iduronidase activity (di Bello *et al.*, 1984). In a modification of the original synthesis of BR1 from D-glucuronolactone (Bashyal *et al.*, 1986, Bashyal, Chow & Fleet, 1987, Bashyal, Chow, Fellows & Fleet, 1987), reduction of the azide 1 afforded a low yield of 2 together with by-products. One of the components of the mixture was crystallized; the structure of this material was determined unequivocally by X-ray crystallographic analysis and shown to be the diketopiperazine 3 (Fig. 2). The absolute stereochemistry was determined by the use of D-glucuronolactone as the starting material. The structure consists of layers of hydrogen bonded molecules running parallel to the *ab* plane (Fig. 3, Fig. 4). Each molecule is a donor and acceptor for 6 hydrogen bonds. Only classical hydrogen bonding was considered.

### Experimental

The title compound was recrystallised by diffusion from a mixture of water and acetonitrile: m.p. 511 K decomposed; [α]_D_^20^ + 29.7 (*c*, 0.35 in H_2_O).

### Refinement

In the absence of significant anomalous scattering, Friedel pairs were merged and the absolute configuration was assigned from the starting material.

The H atoms were all located in a difference map, but those attached to carbon atoms were repositioned geometrically. The H atoms were initially refined with soft restraints on the bond lengths and angles to regularize their geometry (C—H in the range 0.93–0.98, N—H in the range 0.86–0.89 N—H to 0.86 O—H = 0.82 Å) and *U*_iso_(H) (in the range 1.2–1.5 times *U*_eq_ of the parent atom), after which the positions were refined with riding constraints.

One outlying reflection was omitted from the refinement as it was thought to be partially occluded by the beam stop.

### Crystal data

**Table:**

C_12_H_18_N_2_O_8_	*F*(000) = 672
*M**_r_* = 318.28	*D*_x_ = 1.683 Mg m^−^^3^
Orthorhombic, *P*2_1_2_1_2_1_	Mo *K*α radiation, λ = 0.71073 Å
Hall symbol: P 2ac 2ab	Cell parameters from 1646 reflections
*a* = 7.8711 (2) Å	θ = 5–27°
*b* = 8.1526 (2) Å	µ = 0.14 mm^−^^1^
*c* = 19.5783 (5) Å	*T* = 150 K
*V* = 1256.34 (5) Å^3^	Plate, colourless
*Z* = 4	0.40 × 0.10 × 0.10 mm

### Data collection

**Table:**

Nonius KappaCCD area-detector diffractometer	1348 reflections with *I* > 2σ(*I*)
graphite	*R*_int_ = 0.061
ω scans	θ_max_ = 27.5°, θ_min_ = 5.1°
Absorption correction: multi-scan (*DENZO*/*SCALEPACK*; Otwinowski & Minor, 1997)	*h* = −10→10
*T*_min_ = 0.85, *T*_max_ = 0.99	*k* = −10→10
12748 measured reflections	*l* = −25→25
1663 independent reflections	

### Refinement

**Table:**

Refinement on *F*^2^	Primary atom site location: structure-invariant direct methods
Least-squares matrix: full	Hydrogen site location: inferred from neighbouring sites
*R*[*F*^2^ > 2σ(*F*^2^)] = 0.042	H-atom parameters constrained
*wR*(*F*^2^) = 0.101	Method = Modified Sheldrick *w* = 1/[σ^2^(*F*^2^) + (0.06*P*)^2^ + 0.76*P*], where *P* = [max(*F*_o_^2^,0) + 2*F*_c_^2^]/3
*S* = 0.93	(Δ/σ)_max_ = 0.0003
1662 reflections	Δρ_max_ = 0.35 e Å^−^^3^
199 parameters	Δρ_min_ = −0.43 e Å^−^^3^
0 restraints	

### Fractional atomic coordinates and isotropic or equivalent isotropic displacement parameters (Å^2^)

**Table:**

	*x*	*y*	*z*	*U*_iso_*/*U*_eq_	
O1	0.4593 (3)	0.7922 (2)	0.34949 (10)	0.0245	
C2	0.5380 (4)	0.6635 (3)	0.34006 (13)	0.0181	
N3	0.6870 (3)	0.6583 (3)	0.30476 (12)	0.0186	
C4	0.8001 (3)	0.5165 (3)	0.30769 (13)	0.0178	
C5	0.9227 (3)	0.5430 (3)	0.36900 (14)	0.0187	
O6	1.0531 (3)	0.4227 (2)	0.36945 (10)	0.0233	
C7	1.0081 (4)	0.7115 (3)	0.36672 (14)	0.0191	
O8	1.0852 (2)	0.7438 (2)	0.43187 (9)	0.0238	
C9	0.8851 (4)	0.8502 (3)	0.35308 (14)	0.0196	
C10	0.7767 (4)	0.8115 (4)	0.29066 (15)	0.0202	
O11	0.9812 (3)	0.9966 (2)	0.34507 (10)	0.0247	
C12	0.7057 (4)	0.3552 (3)	0.31154 (14)	0.0192	
O13	0.7761 (3)	0.2272 (2)	0.29231 (10)	0.0234	
N14	0.5463 (3)	0.3566 (3)	0.33597 (12)	0.0186	
C15	0.4689 (3)	0.5025 (3)	0.36625 (13)	0.0188	
C16	0.4834 (3)	0.4874 (3)	0.44528 (13)	0.0187	
O17	0.3887 (3)	0.6143 (2)	0.47677 (10)	0.0240	
C18	0.4098 (4)	0.3245 (3)	0.46900 (13)	0.0203	
O19	0.4442 (3)	0.3016 (3)	0.54059 (9)	0.0269	
C20	0.4757 (4)	0.1754 (3)	0.43053 (13)	0.0201	
C21	0.4581 (4)	0.2045 (3)	0.35390 (13)	0.0199	
O22	0.3776 (3)	0.0390 (2)	0.45221 (10)	0.0310	
H41	0.8655	0.5150	0.2656	0.0187*	
H51	0.8535	0.5336	0.4109	0.0227*	
H71	1.0974	0.7116	0.3316	0.0230*	
H91	0.8058	0.8605	0.3927	0.0225*	
H102	0.8459	0.7964	0.2509	0.0230*	
H101	0.6975	0.8990	0.2835	0.0223*	
H151	0.3443	0.5003	0.3557	0.0220*	
H161	0.6054	0.4942	0.4588	0.0216*	
H181	0.2825	0.3283	0.4634	0.0238*	
H201	0.5996	0.1598	0.4417	0.0244*	
H212	0.5077	0.1118	0.3286	0.0232*	
H211	0.3349	0.2163	0.3433	0.0228*	
H81	1.1809	0.7027	0.4365	0.0340*	
H221	0.3488	−0.0299	0.4231	0.0459*	
H191	0.4842	0.3832	0.5589	0.0407*	
H111	0.9114	1.0645	0.3311	0.0377*	
H171	0.4338	0.6804	0.5013	0.0351*	
H61	1.0660	0.3593	0.4021	0.0354*	

### Atomic displacement parameters (Å^2^)

**Table:**

	*U*^11^	*U*^22^	*U*^33^	*U*^12^	*U*^13^	*U*^23^
O1	0.0253 (11)	0.0201 (10)	0.0280 (11)	0.0043 (9)	0.0024 (9)	−0.0012 (8)
C2	0.0189 (14)	0.0226 (14)	0.0128 (13)	0.0006 (13)	−0.0029 (11)	−0.0002 (10)
N3	0.0185 (12)	0.0171 (11)	0.0203 (13)	0.0000 (10)	0.0012 (10)	0.0030 (9)
C4	0.0153 (12)	0.0194 (13)	0.0186 (13)	0.0024 (12)	0.0012 (10)	0.0011 (11)
C5	0.0174 (13)	0.0198 (13)	0.0188 (13)	0.0017 (11)	0.0017 (11)	0.0014 (10)
O6	0.0206 (10)	0.0224 (10)	0.0270 (10)	0.0069 (9)	−0.0006 (9)	0.0050 (9)
C7	0.0193 (14)	0.0209 (13)	0.0170 (13)	0.0003 (11)	0.0006 (11)	−0.0014 (11)
O8	0.0191 (10)	0.0316 (11)	0.0208 (10)	0.0048 (9)	−0.0041 (8)	−0.0056 (8)
C9	0.0191 (14)	0.0192 (14)	0.0205 (14)	−0.0001 (12)	0.0020 (11)	−0.0008 (10)
C10	0.0200 (15)	0.0205 (13)	0.0203 (15)	0.0011 (12)	−0.0010 (11)	0.0004 (11)
O11	0.0265 (10)	0.0169 (10)	0.0307 (10)	0.0000 (10)	−0.0056 (8)	0.0012 (8)
C12	0.0212 (14)	0.0235 (15)	0.0129 (14)	0.0016 (12)	−0.0019 (11)	0.0007 (11)
O13	0.0274 (12)	0.0206 (10)	0.0223 (11)	0.0049 (9)	0.0016 (9)	−0.0027 (8)
N14	0.0182 (12)	0.0180 (12)	0.0197 (12)	−0.0009 (11)	0.0002 (10)	−0.0004 (9)
C15	0.0168 (12)	0.0208 (14)	0.0186 (12)	0.0026 (13)	0.0021 (10)	−0.0015 (12)
C16	0.0151 (12)	0.0214 (14)	0.0197 (12)	0.0014 (12)	0.0006 (10)	−0.0017 (11)
O17	0.0259 (11)	0.0240 (10)	0.0220 (10)	0.0037 (9)	0.0005 (9)	−0.0075 (8)
C18	0.0182 (14)	0.0270 (14)	0.0158 (14)	0.0028 (12)	−0.0007 (11)	0.0015 (11)
O19	0.0392 (12)	0.0261 (10)	0.0153 (9)	0.0003 (10)	−0.0045 (9)	0.0013 (8)
C20	0.0200 (13)	0.0183 (13)	0.0220 (14)	−0.0013 (12)	0.0010 (11)	0.0007 (11)
C21	0.0195 (14)	0.0200 (14)	0.0201 (14)	−0.0034 (12)	0.0015 (12)	−0.0005 (11)
O22	0.0468 (13)	0.0227 (11)	0.0235 (10)	−0.0087 (10)	0.0064 (10)	−0.0005 (8)

### Geometric parameters (Å, °)

**Table:**

O1—C2	1.232 (3)	O11—H111	0.826
C2—N3	1.362 (4)	C12—O13	1.240 (3)
C2—C15	1.510 (4)	C12—N14	1.343 (4)
N3—C4	1.461 (3)	N14—C15	1.462 (3)
N3—C10	1.461 (4)	N14—C21	1.464 (3)
C4—C5	1.555 (4)	C15—C16	1.556 (3)
C4—C12	1.513 (4)	C15—H151	1.002
C4—H41	0.972	C16—O17	1.416 (3)
C5—O6	1.420 (3)	C16—C18	1.521 (4)
C5—C7	1.530 (4)	C16—H161	0.998
C5—H51	0.988	O17—H171	0.804
O6—H61	0.829	C18—O19	1.440 (3)
C7—O8	1.437 (3)	C18—C20	1.521 (4)
C7—C9	1.513 (4)	C18—H181	1.008
C7—H71	0.983	O19—H191	0.819
O8—H81	0.830	C20—C21	1.525 (4)
C9—C10	1.523 (4)	C20—O22	1.419 (3)
C9—O11	1.422 (3)	C20—H201	1.008
C9—H91	1.000	C21—H212	0.984
C10—H102	0.958	C21—H211	0.996
C10—H101	0.957	O22—H221	0.832
			
O1—C2—N3	122.3 (3)	C4—C12—O13	119.8 (3)
O1—C2—C15	120.5 (2)	C4—C12—N14	118.0 (2)
N3—C2—C15	117.1 (2)	O13—C12—N14	122.2 (3)
C2—N3—C4	122.0 (2)	C12—N14—C15	122.7 (2)
C2—N3—C10	119.1 (2)	C12—N14—C21	121.4 (2)
C4—N3—C10	112.9 (2)	C15—N14—C21	113.2 (2)
N3—C4—C5	107.4 (2)	C2—C15—N14	114.8 (2)
N3—C4—C12	113.0 (2)	C2—C15—C16	112.3 (2)
C5—C4—C12	112.8 (2)	N14—C15—C16	108.0 (2)
N3—C4—H41	107.4	C2—C15—H151	107.3
C5—C4—H41	109.1	N14—C15—H151	108.0
C12—C4—H41	106.9	C16—C15—H151	105.9
C4—C5—O6	110.9 (2)	C15—C16—O17	109.7 (2)
C4—C5—C7	112.0 (2)	C15—C16—C18	110.2 (2)
O6—C5—C7	107.6 (2)	O17—C16—C18	107.7 (2)
C4—C5—H51	106.8	C15—C16—H161	109.3
O6—C5—H51	109.8	O17—C16—H161	110.5
C7—C5—H51	109.7	C18—C16—H161	109.5
C5—O6—H61	121.6	C16—O17—H171	121.1
C5—C7—O8	108.9 (2)	C16—C18—O19	109.8 (2)
C5—C7—C9	113.3 (2)	C16—C18—C20	114.6 (2)
O8—C7—C9	106.9 (2)	O19—C18—C20	108.3 (2)
C5—C7—H71	109.6	C16—C18—H181	108.6
O8—C7—H71	108.6	O19—C18—H181	107.2
C9—C7—H71	109.5	C20—C18—H181	108.0
C7—O8—H81	114.0	C18—O19—H191	113.2
C7—C9—C10	110.2 (2)	C18—C20—C21	109.4 (2)
C7—C9—O11	107.8 (2)	C18—C20—O22	107.0 (2)
C10—C9—O11	112.5 (2)	C21—C20—O22	111.5 (2)
C7—C9—H91	109.0	C18—C20—H201	108.8
C10—C9—H91	106.9	C21—C20—H201	108.8
O11—C9—H91	110.3	O22—C20—H201	111.2
C9—C10—N3	107.2 (2)	C20—C21—N14	108.9 (2)
C9—C10—H102	111.1	C20—C21—H212	109.8
N3—C10—H102	108.5	N14—C21—H212	110.0
C9—C10—H101	109.1	C20—C21—H211	107.9
N3—C10—H101	110.5	N14—C21—H211	109.3
H102—C10—H101	110.3	H212—C21—H211	110.9
C9—O11—H111	104.2	C20—O22—H221	118.2

### Hydrogen-bond geometry (Å, °)

**Table:**

*D*—H···*A*	*D*—H	H···*A*	*D*···*A*	*D*—H···*A*
C4—H41···O11^i^	0.97	2.48	3.455 (4)	176
C15—H151···O6^ii^	1.00	2.39	3.337 (4)	157
O8—H81···O17^iii^	0.83	1.95	2.756 (4)	162
O22—H221···O1^iv^	0.83	2.22	2.917 (4)	141
O19—H191···O11^v^	0.82	2.12	2.793 (4)	139
O11—H111···O13^vi^	0.83	1.86	2.685 (4)	173
O17—H171···O8^v^	0.80	1.87	2.633 (4)	157
O6—H61···O19^vii^	0.83	1.97	2.680 (4)	143

Symmetry codes: (i) −*x*+2, *y*−1/2, −*z*+1/2; (ii) *x*−1, *y*, *z*; (iii) *x*+1, *y*, *z*; (iv) *x*, *y*−1, *z*; (v) *x*−1/2, −*y*+3/2, −*z*+1; (vi) *x*, *y*+1, *z*; (vii) *x*+1/2, −*y*+1/2, −*z*+1.
